# Surface Pre-Reacted Glass Filler Contributes to Tertiary Dentin Formation through a Mechanism Different Than That of Hydraulic Calcium-Silicate Cement

**DOI:** 10.3390/jcm8091440

**Published:** 2019-09-11

**Authors:** Motoki Okamoto, Manahil Ali, Shungo Komichi, Masakatsu Watanabe, Hailing Huang, Yuki Ito, Jiro Miura, Yujiro Hirose, Manabu Mizuhira, Yusuke Takahashi, Daisuke Okuzaki, Shigetada Kawabata, Satoshi Imazato, Mikako Hayashi

**Affiliations:** 1 Department of Restorative Dentistry and Endodontology, Osaka University Graduate School of Dentistry, 1-8, Yamadaoka, Suita, Osaka 565-0871, Japan; 2 Division for Interdisciplinary Dentistry, Osaka University Dental Hospital, 1-8 Yamadaoka, Suita, Osaka 565-0871, Japan; 3 Department of Oral and Molecular Microbiology, Osaka University Graduate School of Dentistry, 1-8, Yamadaoka, Suita, Osaka 565-0871, Japan; 4 Bruker Japan K.K. Nano Analytics Division, 3-9 Moriyacho, Yokohama, Kanagawa 221-0022, Japan; 5 Genome Information Research Center, Research Institute for Microbial Diseases, Osaka University, 1-1, Yamadaoka, Suita, Osaka 565-0871, Japan; 6 Department of Biomaterials Science, Osaka University Graduate School of Dentistry, 1-8, Yamadaoka, Suita, Osaka 565-0871, Japan

**Keywords:** direct pulp capping, surface pre-reacted glass filler, µCT, µXRF, RNA sequence

## Abstract

The induction of tissue mineralization and the mechanism by which surface pre-reacted glass-ionomer (S-PRG) cement influences pulpal healing remain unclear. We evaluated S-PRG cement-induced tertiary dentin formation in vivo, and its effect on the pulp cell healing process in vitro. Induced tertiary dentin formation was evaluated with micro-computed tomography (μCT) and scanning electron microscopy (SEM). The distribution of elements from the S-PRG cement in pulpal tissue was confirmed by micro-X-ray fluorescence (μXRF). The effects of S-PRG cement on cytotoxicity, proliferation, formation of mineralized nodules, and gene expression in human dental pulp stem cells (hDPSCs) were assessed in vitro. μCT and SEM revealed that S-PRG induced tertiary dentin formation with similar characteristics to that induced by hydraulic calcium-silicate cement (ProRoot mineral trioxide aggregate (MTA)). μXRF showed Sr and Si ion transfer into pulpal tissue from S-PRG cement. Notably, S-PRG cement and MTA showed similar biocompatibility. A co-culture of hDPSCs and S-PRG discs promoted mineralized nodule formation on surrounding cells. Additionally, S-PRG cement regulated the expression of genes related to osteo/dentinogenic differentiation. MTA and S-PRG regulated gene expression in hDPSCs, but the patterns of regulation differed. S-PRG cement upregulated *CXCL-12* and *TGF-β1* gene expression. These findings showed that S-PRG and MTA exhibit similar effects on dental pulp through different mechanisms.

## 1. Introduction

Conventional glass-ionomer cement (Co-GIC) is set through an acid-base reaction of fluoroaluminosilicate glass and poly-acrylic acid solution. It was developed by Wilson and Kent [1,2] and has become one of the most widely applied materials in the dental clinic. Co-GIC is typically applied as an atraumatic restorative treatment (ART), as minimal interventions have been recommended by the World Health Organization (WHO). They have advocated removing carious lesions using simple hand instruments and using Co-GIC as a biocompatible material for filling [3].

Vital pulp therapy using a bioactive material is an important approach to preserve tooth vitality and function, as it helps to provide protection against harmful stimuli [4,5] and supply effective stimulators that have a regenerative effect [6]. An ideal pulp capping material should have antibacterial activity to provide an ideal biological environment for dental pulp tissue repair. Nevertheless, the mechanisms of wound healing in pulpal tissue remain unclear. Calcium hydroxide (Ca(OH)_2_) pulp capping material has been used as the gold standard pulp capping agent [7]. However, Ca(OH)_2_ has disadvantages such as inducing coagulation, necrosis, pathologic calcification, and pulp chamber obliteration [8,9]. ProRoot mineral trioxide aggregate (MTA) (‘MTA’: Dentsply Sirona, Ballaigues, Switzerland), a hydraulic cement that contains calcium silicate as a main component, has been used by the clinician for various regenerative therapies, notably, it induces greater dentin bridge formation than conventional Ca(OH)_2_ material [10,11]. However, MTA also has limitations, including its high cost, the potential to release hazardous substances, tooth discoloration, prolonged setting time, mixture inconsistency, handling inconvenience, and inability to adhere to the dentin—all of which limit its application in dental procedures [12]. Additionally, Co-GIC cannot be considered as a direct pulp capping material due to its lack of biocompatibility when directly applied to exposed pulp [13].

Surface pre-reacted glass-ionomer (S-PRG) fillers are prepared by an acid–base reaction of fluoroboroaluminosilicate glass and poly-acrylic acid solution [14]. S-PRG filler has been demonstrated to protect against the demineralization of enamel and dentin [15,16]; moreover, it has a mineral inductive effect [14] and decreases plaque formation [17]. This filler has already been adopted into various dental materials, including resin composites [18] and root canal sealers [19]. S-PRG filler releases multiple ions, including fluoride, strontium, sodium, borate, aluminum, and silicate ions [20], which may provide various biological reactions. For example, fluoride and borate ions have antimicrobial effects [21], strontium ions enhance bone formation [22,23], and silicate ions enhance bone formation [24]. We have previously reported that S-PRG fillers induce tertiary dentin formation with matrix collagen in demineralized tissue samples when they were applied as a direct pulp capping cement in rat models [25]. Recently, we have also modified S-PRG fillers by incorporating lithium ions into them that could promote reparative dentin formation when applied on exposed pulp surface by activating the Wnt signaling pathway [26]. However, a thorough evaluation of the induction of mineralized tissue beneath the pulp capping material and the biological role of S-PRG cement for pulpal repair has yet to be performed.

Therefore, the present study evaluated the quality and quantity of tertiary dentin formation induced by S-PRG cement in rat teeth, using micro-computed tomography (μCT) and microstructural analysis with scanning electron microscopy (SEM). The distribution of the releasing ions related to the mineralized structure that was formed by S-PRG cement was evaluated using micro-X-ray fluorescence (µXRF). To elucidate the mechanisms by which tertiary dentin formation is induced, the effects of S-PRG cement on pulpal repair function, proliferation, cytotoxicity, formation of mineralized nodules, and gene expression were analyzed using a cell counting test, lactate dehydrogenase (LDH) assay, alizarin red staining, SEM, μXRF, RNA sequencing, and real-time polymerase chain reaction (PCR), respectively.

## 2. Materials and Methods

### 2.1. Ethical Statement

The animal experimental protocol in this study was approved by the Ethical Guidelines Committee for Animal Care of Osaka University Graduate School of Dentistry (No. 28-013-0). All surgeries were performed under general anesthesia, and all efforts were taken to minimize pain or discomfort.

### 2.2. Cell Culture

We purchased human dental pulp stem cells (hDPSCs) collected from human sound third molars (Lonza, Basel, Switzerland). These cells have been confirmed to be cell populations containing stem cells. Surface antigens on these cells have been confirmed for Cluster Designation (CD) 105+, CD166+, CD29+, CD90+, CD73+, CD133-, CD34-, CD45- by flow cytometry. Cells were detached (TrypLE Select, Life Technologies, Carlsbad, CA, USA) and sub-cultured when they reached 80% confluence. For the following assay, hDPSCs (passages 2–5) were cultured in α-Minimum Essential Media (α-MEM; Life Technologies, Carlsbad, CA, USA) supplemented with penicillin-streptomycin 10 μg/mL (Sigma-Aldrich, St. Louis, MO, USA). In the in vitro assays, 1% fetal bovine serum (FBS; Sigma-Aldrich, St. Louis, MO, USA) was used.

### 2.3. Cement Components and Material Preparation

Experimental S-PRG cement and Co-GIC (Base cement) were obtained from Shofu (Kyoto, Japan). White ProRoot MTA (WMTA; Dentsply Sirona, Ballaigues, Switzerland) was used for comparison purposes. Table 1 presents the composition of each material. Co-GIC and ProRoot MTA were mixed in accordance with the manufacturer’s protocol. S-PRG cement was made by manual mixing powder and liquid (powder/liquid = 1.5/1 in weight). Each cement was then placed in a polytetrafluoroethylene mold (diameter of 3 mm and thickness of 1 mm) and pressed between two glass slides. Discs were incubated at 37 °C in a humidified chamber for 24 h. Fresh discs were sterilized with Ethylene Oxide Gas (EOG) before inserting them into the plates.

### 2.4. LDH Cell Cytotoxicity Assay

The biocompatibility of S-PRG cements to hDPSCs was determined using the LDH cytotoxicity assay (Thermo Fisher Scientific, Waltham, MA, USA) in accordance with the manufacturer’s protocol. Briefly, 3.0 × 10^6^ hDPSCs were seeded in a 35 mm dish containing a cell culture medium with one S-PRG cement, MTA, or Co-GIC disc. A group containing no disc was used as the control. Following incubation time points (1, 2, 3, 5, 7, and 14 days), the supernatant was collected from each dish and centrifuged at 250 g for 5 min. The supernatant was then added to an LDH agent and incubated for 30 min away from light. The absorbance of the LDH reaction was measured at 405 nm using a microplate reader (ARVO MX, PerkinElmer, Waltham, MA, USA). Eight wells were measured for each supernatant group.

### 2.5. Cell Proliferation Assay

To evaluate the effects of S-PRG cement on hDPSC growth, cell counting was performed. The collected hDPSCs (1.0 × 10^6^ cells) were cultured in α- Minimum Essential Media (MEM) supplemented with 1% FBS and S-PRG cement, MTA, or Co-GIC discs. Cell proliferation was evaluated using the trypan blue exclusion method following 3 and 5 days of culturing. Cell morphology after treatment was also observed under light microscopy (ECLIPSE CI-L, Nikon, Tokyo, Japan) 7 days post culturing.

### 2.6. Pulp Capping Procedure

Nine Wistar rats weighing 200–230 g (CLEA Japan Inc., Tokyo, Japan) were used for the in vivo experiments. Ten maxillary first molars were randomly divided into two groups: 1 week and 4 weeks groups, respectively. Each group was subdivided into another two groups according to the capping materials used at each experiment. One week group samples were used to evaluate the distribution of ion release from materials (*n* = 3 per group) using micro-X-ray fluorescence spectroscopy (µXRF). The four week group specimens were assessed for mineralized tissue formation (*n* = 8 per group) using micro-computed tomography and scanning electron microscope.

The pulp capping procedure was performed as previously described [25]. Briefly, anesthesia was induced with 0.3 mg/kg medetomidine hydrochloride (Domitol; Meiji Seika Pharma Co., Ltd., Tokyo, Japan), 4.0 mg/kg midazolam (Dormicum; Astellas Pharma Inc., Tokyo, Japan), and 5.0 mg/kg butorphanol (Vetorphale; Meiji Seika Pharma Co., Ltd., Tokyo, Japan). Then, a 0.5 mL injection of 2% lidocaine with 1:100,000 epinephrine (Xylocaine; Dentsply Pharmaceuticals Inc., York, PA, USA) was administered as a local infiltrative anesthesia, and the experimental teeth were cleaned and isolated with a rubber dam sheet (Heraeus Kulzer, South Bend, IN, USA) with a custom-made rubber dam clamp (YDM, Tokyo, Japan) to perform treatment in a sterilized environment. A class-I cavity was prepared on the occlusal surface with pulp exposure using a steel #1 round bur (diameter of 0.8 mm; Dentsply Sirona) by one experienced operator under a sterile saline spray to establish a stable and standardized cavity (diameter and depth of approximately 1 mm). The preparation time was approximately 10 s for each sample.

Subsequently, the exposed pulp was directly capped with MTA or S-PRG cement after being mixed in accordance with the manufacturer’s protocol. To ensure sealing of the capped sites, cavities were restored with glass-ionomer cement (Fuji IX; GC International Corp., Tokyo, Japan).

### 2.7. Distribution of Released Ions from S-PRG Cement Using µXRF Analysis 

The one week group’s rats were sacrificed and targeted teeth were collected and chemically fixed in 2% paraformaldehyde and 2.5% glutaraldehyde, followed by graded ethanol series dehydration. Samples were embedded in epoxy resin (Quetol-812, Nissin EM, Tokyo, Japan). Then they were prepared in 4 mm^3^ cubes. Resin samples were sectioned in the sagittal axis, and the surfaces were polished and observed under µXRF spectrometer (M4 TORNADO, Bruker, Berlin, Germany). 

Elemental maps were produced for calcium (Ca), phosphorus (P), strontium (Sr), aluminum (Al), silicon (Si), and bismuth (Bi) in vivo and Ca and P in vitro based on the resulting data obtained. The basic settings parameters of the µXRF were as follows: voltage, 50 kV; current, 600 µA; pixel size, 4 µm; and exposure time, ~12 min in dry conditions. Esprit software (Bruker) was used to conduct elemental settings that displayed the distributions of these elements according to their intensities beneath the pulp capping area. 

### 2.8. Microstructure Analysis Using Scanning Electron Microscopy

The maxillae of the 4 week rats were dissected and fixed in 2% paraformaldehyde and 2.5% glutaraldehyde at approximately 25 °C for 4 h. After µCT scanning, the same specimens were used for SEM evaluation. The tissue specimens were dehydrated in ascending concentrations of ethanol, embedded in epoxy resin (Quetol-812, Nissin EM), and surfaces were polished using diamond abrasive sheets (granularity 30 μm–1 μm, Maruto instrument co., LTD, Tokyo, Japan). The sample surfaces were coated with platinum (JEC-3000F, JEOL, Tokyo, Japan). Backscattered electron images were obtained with SEM (JSM-6510V).

### 2.9. Three-Dimensional µCT Analyses

The animals were sacrificed at 4 weeks after undergoing direct pulp capping. The induced tertiary dentin was analyzed using a µCT scanner (R-mCT2; Rigaku, Tokyo, Japan) with a scanning resolution of 10 μm intervals in an individual image, as previously described [27]. The basic scanner parameters were as follows: voltage, 90 kV; current, 160 µA; and exposure time, ~3 min in dry conditions. After scanning, 512 consecutive tomographic slice images were obtained. The image data were then reconstructed using three-dimensional reconstruction imaging software TRI/3D-BON (Ratoc System Engineering, Tokyo, Japan). A calibration curve was prepared from the standard mineral reference phantom (Ratoc System Engineering), converted to degrees of calcification (mg/cm^3^); additionally, the degree of tooth mineralization was measured. To calibrate the mineral density measurement, the mineral reference phantom was scanned prior to all experimental specimens. Each region of interest (ROI) was analyzed based on the tertiary dentin volume (DV) and tertiary dentin mineral density (DMD).

### 2.10. Mineralized Nodule Formation Assay

To evaluate the effects of S-PRG cement on hDPSCs, a mineralized nodule formation assay was performed. After the collected hDPSCs reached subconfluence when cultured in α-MEM supplemented with 1% FBS, a material disc of S-PRG cement or MTA was added to each cell dish. The mineralized nodules formed around each material sample were evaluated by light microscopy, SEM and μXRF. 

### 2.11. RNA Extraction and Quantitative Real-Time Polymerase Chain Reaction

hDPSCs were seeded in 35 mm cell culture plates at a density of 30 × 10^6^ cells and 1% FBS supplemented culture media with an S-PRG cement disc or MTA disc for 7 days. Untreated hDPSCs were used as a control. The total RNA was collected using the RNeasy Mini RNA isolation kit (Qiagen N.V, Hilden, Germany), and was used as a template for cDNA synthesis in accordance with the manufacturer’s recommendations. RNA concentrations were calculated from the absorption ratio (OD260/OD280) using the NanoDrop system (Thermo Fisher Scientific). cDNA was synthesized using ReverTra Ace qPCR RT Master Mix with gDNA Remover (Toyobo, Osaka, Japan) in accordance with the manufacturer’s instructions. Real-time PCR was performed using SYBR Green PCR Master Mix (Applied Biosystems, Foster City, CA, USA), and fluorescence was detected using the ABI 7500 Fast system (Applied Biosystems). Genes related to pulp wound healing, including *CXCL-12* and *TGF-β1*, were amplified using the custom-made forward and reverse primers listed in Table 2. The data were normalized against an internal control, glyceraldehyde-3-phosphate dehydrogenase (*GAPDH*).

### 2.12. RNA Sequencing and Data Analysis

The hDPSCs were seeded in 35 mm cell culture plates at a density of 30 × 10^6^ cells and in 1% FBS media with an S-PRG cement specimen and MTA specimen in the culture media for 7 days. Untreated hDPSCs were used as a control. Total RNA was prepared using the RNeasy Mini RNA isolation kit (Qiagen, N.V, Hilden, Germany) for RNA sequence analysis. Total RNAs from three independent trials were collected for each group. RNA integrity was assessed with a 2100 bioanalyzer (Agilent Technologies, Santa Clara, CA, USA). Directional RNA-Seq libraries were created using the TruSeq stranded mRNA sample Prep Kit (Illumina Inc., San Diego, CA, USA), in accordance with the manufacturer’s recommendations. Library sequencing was carried out using the Illumina HiSeq 2500 system with 75 bp single-end reads. Raw reads were deposited into the DNA Deta Bank of Japan (DDBJ) sequence read archive (DRA, accession number: DRA008317). Data were generated in the standard Sanger FastQ format and phred-type quality scores Q30 were used for quality trimming. RNA-Seq reads were mapped to the GRCh38/hg38 human assembly, using the commercially available CLC Genomics workbench (version 9.5.2, CLC bio, Aarhus, Denmark). Reads counts and fragments per kilobase of gene per million read values were used for heatmap visualization with integrated Differential Expression and Pathway analysis (iDEP, PMID: 30567491) and ClustVis (PMID: 25969447), respectively.

### 2.13. Statistical Analysis

Significant differences between two groups and multiple groups were evaluated by Student’s *t*-test or one-way ANOVA with Tukey’s post hoc test, respectively. *p* values < 0.05 were considered to be statistically significant. 

## 3. Results

### 3.1. Microstructure Analysis Using Scanning Electron Microscopy

**Figure 1:**
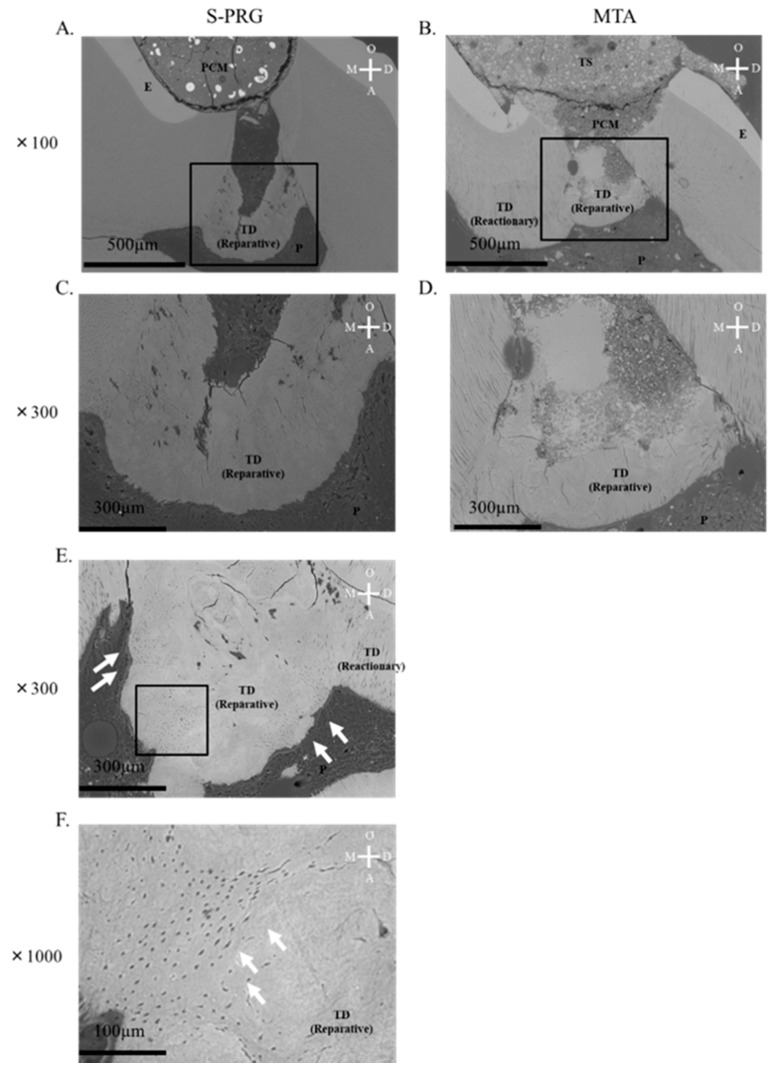
Scanning electron microscopy results after direct pulp capping in 4 week samples. (**A**) representative mineralized tissue image is shown for each material. Both surface pre-reacted glass-ionomer cement (S-PRG) and mineral trioxide aggregate (MTA) induced tertiary dentin (**A**–**D**); high magnification image in (**C**,**D**). Tertiary dentin with tubular structure was confirmed in most S-PRG samples (**E**). White arrow shows tubular structure (**F**). PCM = pulp capping material, T.S = temporary sealing, P = pulp, T.D = tertiary dentin, D = distal, M = mesial, O = occlusal, and A = apical.

Representative SEM images of tertiary dentin induced by S-PRG cement and MTA after 4 week pulp capping are shown in Figure 1. Tertiary dentin formation was induced by each material beneath pulpal tissue. Reactionary and reparative dentin was formed after pulp capping using each material. Tubular structures were observed in the reparative dentin induced by the S-PRG cement (Figure 1E,F; white arrows). Each material induced tertiary dentin formation in the pulpal tissue and showed similar effects in the microstructural evaluation.

### 3.2. Three-Dimensional μCT Analysis of Tertiary Dentin Formation

**Figure 2:**
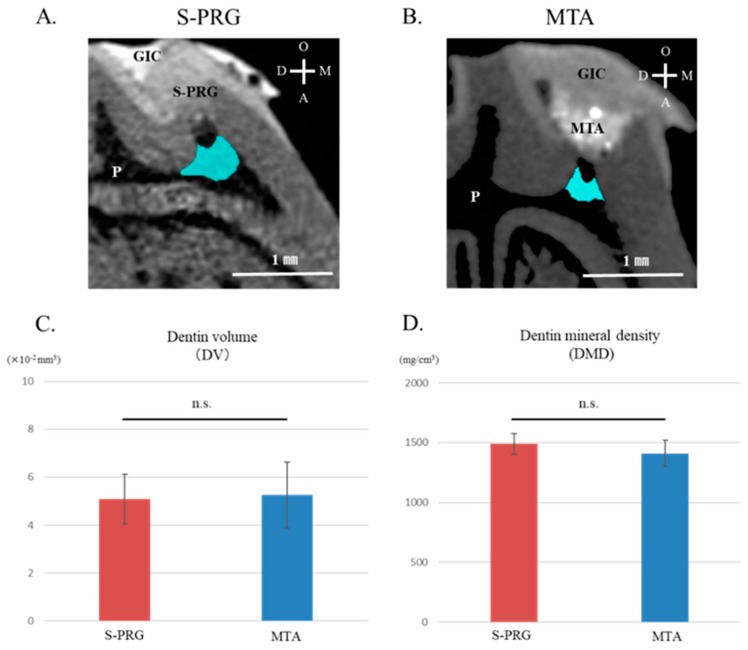
Micro-computed tomography (µCT) results after direct pulp capping in 4 week samples. Representative sagittal µCT images of tertiary dentin induced by surface pre-reacted glass-ionomer (S-PRG) and mineral trioxide aggregate (MTA) are shown (**A**,**B**). Tertiary dentin formation induced by S-PRG and MTA show similar characteristics in dentin volume (DV) (**C**) and dentin mineral density (DMD) (**D**). Data represent mean ± standard deviation (*n* = 8). GIC = glass-ionomer cements, PA = Pulp capping agent, P = pulp, T.D = Tertiary dentin, D = distal, M = mesial, O = occlusal, A = apical, and n.s. = not significant (*p* > 0.05).

Representative μCT images of tertiary dentin induced by S-PRG cement and MTA are shown as light blue areas in Figure 2A,B. Tertiary dentin formation was confirmed in all specimens using each material. S-PRG cement and MTA showed similar abilities to increase tertiary DV beneath the injured site (Figure 2C). There was no significant difference in tertiary DMD values between S-PRG cement and MTA (Figure 2D). 

### 3.3. Distribution of Releasing Ions from S-PRG Cement Using µXRF Analysis

**Figure 3:**
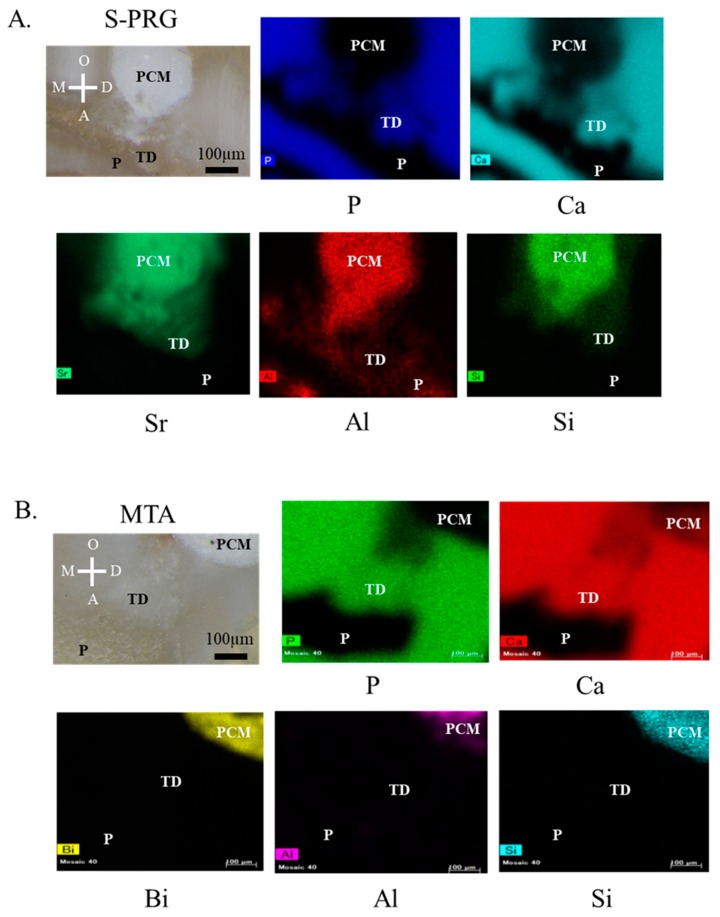
Micro-X-ray fluorescence (µXRF) results after direct pulp capping in 1 week samples. Representative sagittal µXRF of tertiary dentin induced by surface pre-reacted glass-ionomer (S-PRG) and mineral trioxide aggregate (MTA) is shown (**A**,**B**). Optical microscopic image and element mapping images are shown (**A**,**B**). Mineralized areas of tertiary dentin can be distinguished by the high content of calcium (Ca) and phosphorus (P), which are the main constituents of hydroxyapatite in each material. Strontium and silicate were detected in the pulpal tissue beneath the S-PRG cement. T.D = Tertiary dentin, PCM = pulp capping material, P = pulp, D = distal, M = mesial, O = occlusal, and A = apical.

Elemental setting revealed the distribution of the ions released from the S-PRG cement according to their intensities in the scanned area following pulp capping, using material from 1 week samples (Figure 3A,B). The following elements were identified: Ca, P, Sr, Al, Si, and Bi. The areas generating tertiary dentin could be distinguished by the high content of Ca and P, which are the main constituents of hydroxyapatite (Ca_10_(PO_4_)_6_(OH)_2_). Sr, Si, and Al released from S-PRG cement were present beneath the pulp capping materials (Figure 3A). In the MTA samples, the mineralized areas could be distinguished by the high content of Ca and P. Although Bi, Al, and Si ions constituting MTA were confirmed, these ions were not presented in the pulp (Figure 3B). 

### 3.4. LDH Cytotoxicity and Proliferation Assay

**Figure 4:**
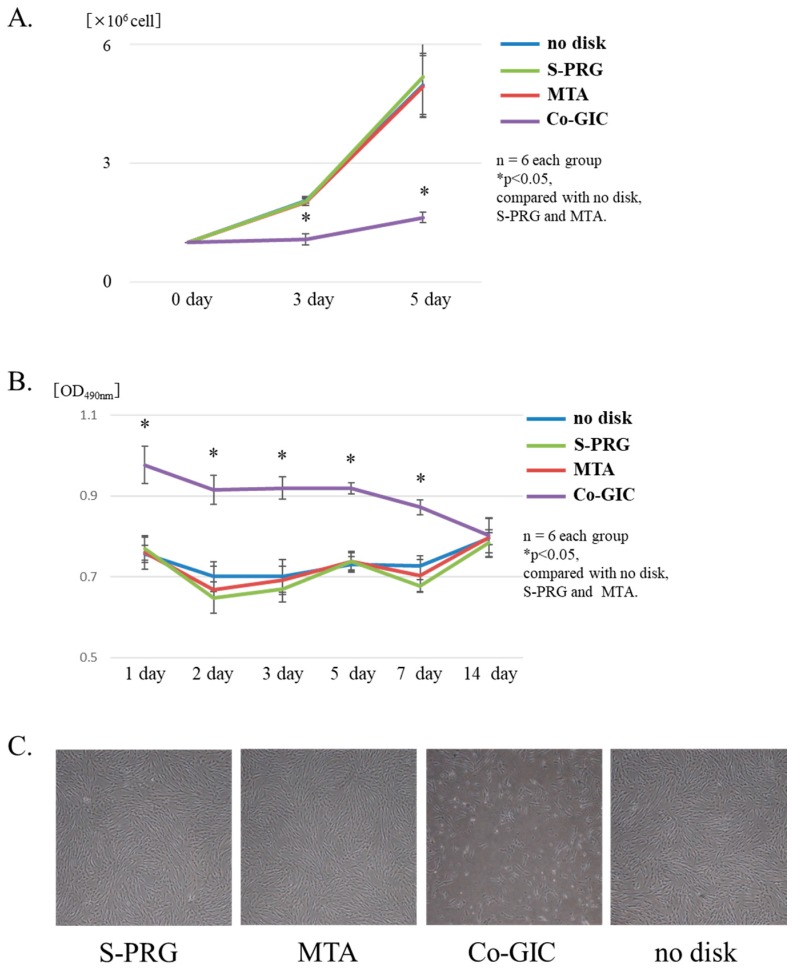
Comparison of the biocompatibility of each material disc co-cultured with human dental pulp stem cells (hDPSCs). Proliferation results are shown in **A**. Conventional glass-ionomer cement (Co-GIC) inhibited hDPSC proliferation (*p* < 0.05). Surface pre-reacted glass-ionomer (S-PRG) cement and mineral trioxide aggregate (MTA) did not affect cell proliferation. Cell cytotoxicity was evaluated using the lactate dehydrogenase (LDH) assay in **B**. Co-GIC showed cytotoxicity for 7 days (*p* < 0.05). S-PRG and MTA showed high biocompatibility at all time points. Cell morphology was spindle-shaped and similar among S-PRG, MTA, and the control (**C**,**D**,**F**). No differences in morphology and no floating cells were observed (**C**,**D**,**F**) under light microscopy. Co-GIC inhibited cell proliferation and many floating cells were observed (**E**). Data represent mean ± standard deviation (*n* = 8).

The effects of cell proliferation on hDPSCs were evaluated using a cell counting assay at 3 days and 5 days. Compared to the control, S-PRG cement and MTA increased hDPSC proliferation and showed similar effects. The Co-GIC group significantly inhibited cell proliferation when compared to that in the other groups (*p* < 0.05, Figure 4A). The cytotoxicity (up to 14 days for each material disc) was compared with that of the control group. Significantly increased cytotoxicity was observed in the Co-GIC group when compared with that in the other groups (*p* < 0.05, Figure 4B). The cytotoxicity of Co-GIC could not be confirmed after 14 days. S-PRG cement and MTA exhibited a similar tendency for hDPSC proliferation throughout the culture period when compared with that of the control. Under microscopic observation, the hDPSCs cultured with S-PRG cement and MTA were spindle-shaped. There were no differences in morphology or floating cells observed (Figure 4C,D). Based on these findings, Co-GIC was considered an unsuitable experimental control for conducting subsequent experiments on Co-GIC, in which powder components differed from S-PRG cement.

### 3.5. Mineralized Nodule Formation Assay

**Figure 5:**
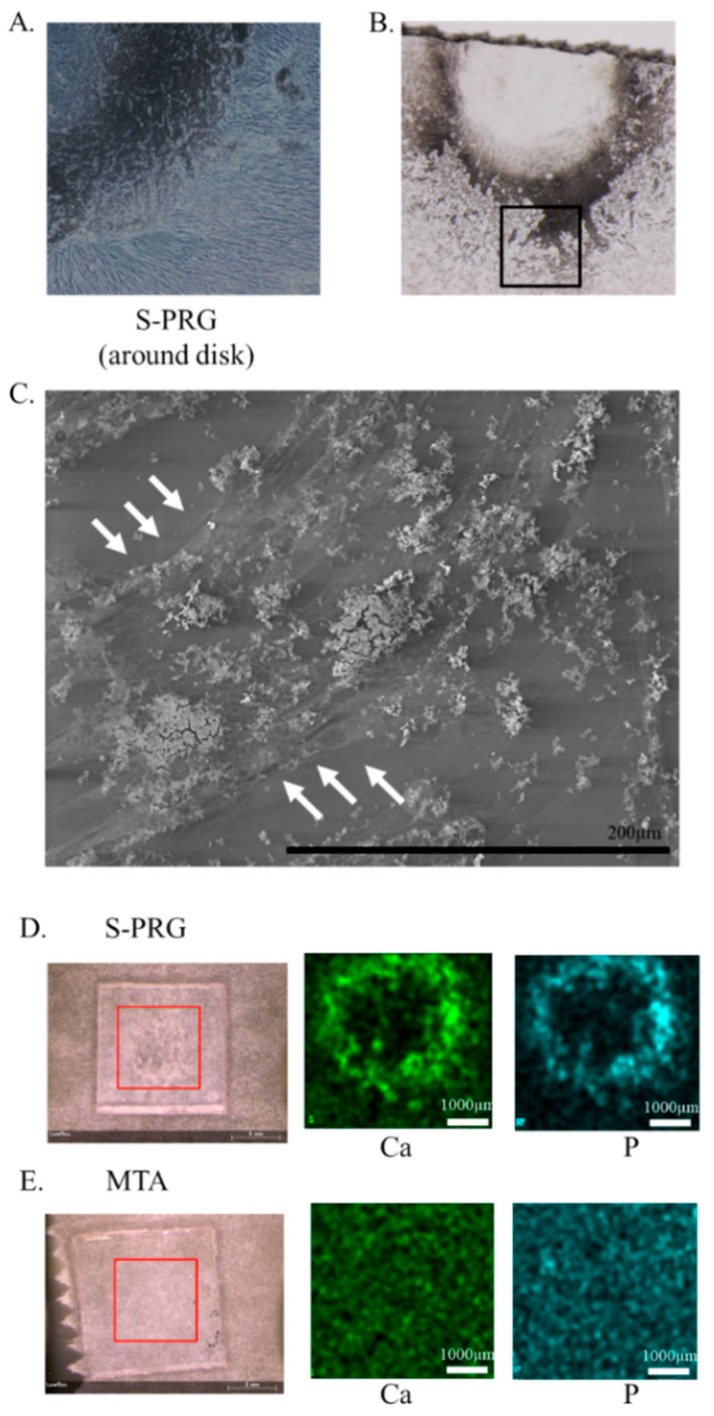
The effect of mineralized nodule formation on human dental pulp stem cells (hDPSCs) observed using scanning electron microscopy (SEM) and micro-X-ray fluorescence (μXRF). Precipitation was confirmed around the surface pre-reacted glass-ionomer (S-PRG) disc (**A**,**B**), but not around the mineral trioxide aggregate (MTA) or control disc at 7 days. No precipitate was observed after 7 days in co-culture with MTA and S-PRG cement without hDPSCs. SEM image depicts the black box area in (**B**). Precipitates were observed using SEM; these were consistent with the locations of hDPSCs and were absent from cell-free sites (**C**). Representative μXRF images of S-PRG and MTA are shown in (**D**,**E**). The following elements were identified: calcium (Ca) and phosphorus (P) around the S-PRG disc.

The effect of mineralized nodule formation on hDPSCs was evaluated using light microscopy, SEM, and μXRF assays without osteoinductive media at 7 days. After co-culture with an S-PRG cement disc, precipitates due to ion release were confirmed from S-PRG cement around the disc. Precipitates and cells were in close contact with each other (Figure 5A,B), as observed using SEM. The precipitates were not present at cell-free sites (Figure 5C). No precipitates were observed after 7 days of co-culture with MTA and S-PRG cement without hDPSCs. Representative μXRF findings of S-PRG cement and MTA are shown in Figure 5D,E. The following elements were identified: Ca and P around the S-PRG disc. This result showed that precipitate around S-PRG cement was derived from hDPSCs, rather than nonspecific precipitation, because these elements were not released from S-PRG cement. 

### 3.6. RNA Sequence Analysis

**Figure 6:**
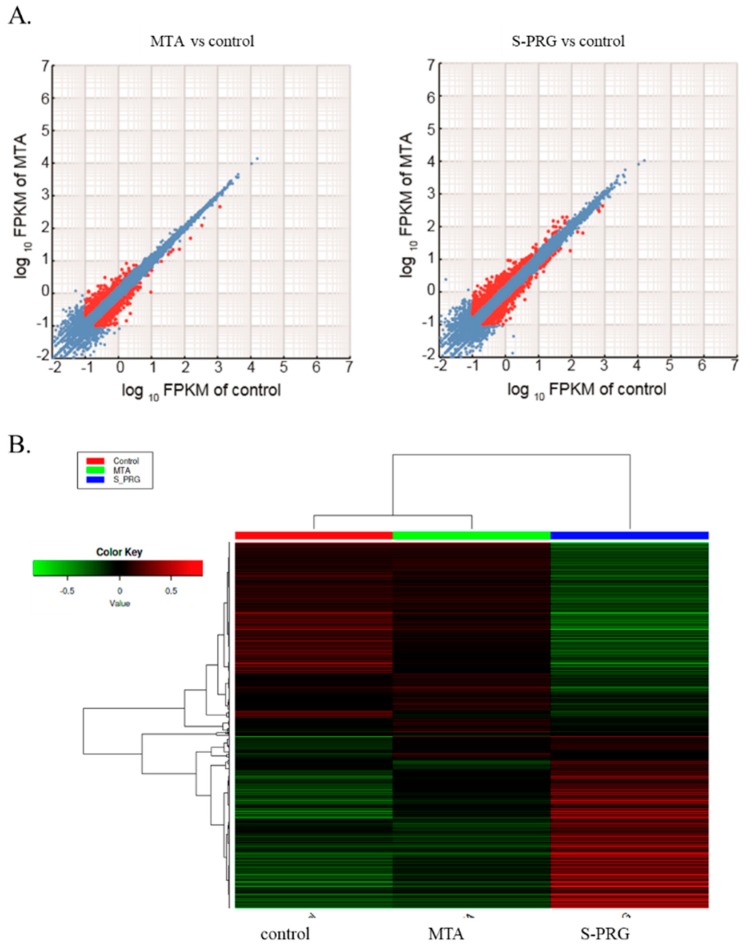
RNA-Seq global reports. Scatter plots comparing global gene expression patterns between mineral trioxide aggregate (MTA) and control, as well as between surface pre-reacted glass-ionomer (S-PRG) and control (**A**). Heat map showing clustering of the 1000 most variable genes expressed in all samples (**B**). Each column represents one sample, and each row represents one gene. Hierarchical clustering was illustrated using the average linkage method with correlation distance. Color coding is based on empirical analysis of digital gene expression data in R (edgeR) log-transformed read count values. The color key indicates Z-scores that display the relative values of all tiles within all samples; green indicates lowest expression, black indicates intermediate expression, and red indicates highest expression.

**Figure 7:**
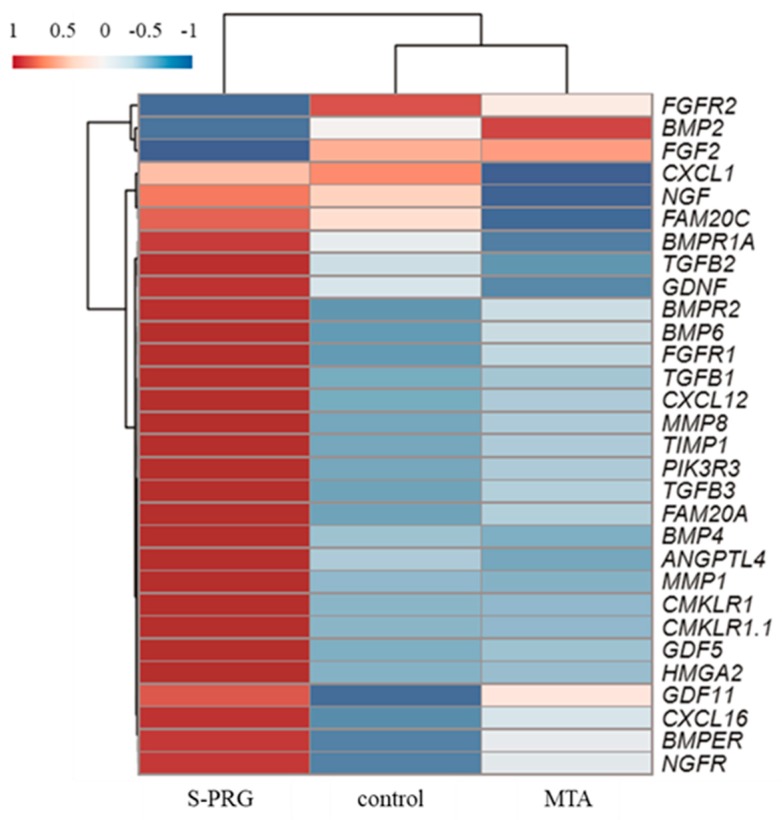
Heat map of key transcripts for the osteo/dentinogenic differentiation process. Fragments per kilobase of gene per million reads (FPKM) values were used for heatmap visualization. FPKM values were collected from genes involved in the osteo/dentinogenic differentiation process. The heatmap was visualized by use of the web tool ClustVis (http://biit.cs.ut.ee/clustvis/) with default parameters. Red and blue indicate induced and repressed genes, respectively.

The effect of S-PRG cement on hDPSC global gene expression was evaluated by RNA sequencing. Scatter plots comparing global gene expression patterns between MTA and control, as well as between S-PRG cement and control, are shown in Figure 6A. Red dots indicate genes up-or down-regulated by at least 2-fold in each dataset. This result demonstrated that both S-PRG cement and MTA influenced gene expression in hDPSCs. Heat map of the clustering of the 100 most variable genes expressed in all samples is demonstrated in Figure 6B. Each column represents a sample, and each row represents a gene. These results showed that MTA and S-PRG cement regulated gene expression in hDPSCs but had different patterns of regulation. A heat map of the key transcripts for the osteo/dentinogenic differentiation process is shown in Figure 7. These genes were consistent with those identified in previous reports related to osteo/dentinogenic differentiation [28,29,30,31,32,33,34,35]. 

### 3.7. Real-Time PCR Analysis of Pulpal Wound Healing Process-Related Genes

**Figure 8:**
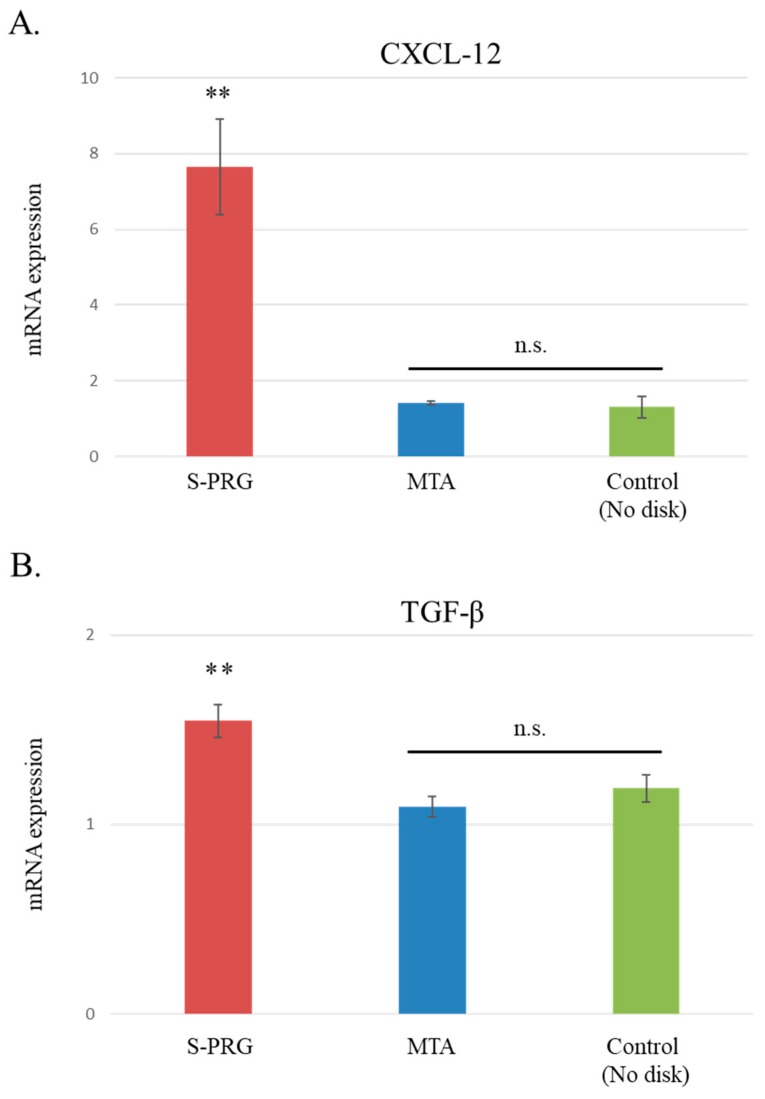
Gene expression normalized with reference to that of the housekeeping gene, *GAPDH*. *CXCL-12* and *TGF-β1* gene levels were significantly elevated in the surface pre-reacted glass-ionomer (S-PRG) disc group, when compared with the mineral trioxide aggregate (MTA) and control groups after the 7-day culture (*p* < 0.01). Data represent mean ± standard deviation (*n* = 6). ** *p* < 0.01, S-PRG versus MTA and control. n.s. = not significant (*p* > 0.05).

The effects of S-PRG cement on hDPSC gene expression related to wound healing were evaluated. After co-culturing hDPSCs with each material disc for 7 days, real-time PCR was performed to confirm the results of RNA sequencing and to measure the gene expression levels of *CXCL-12* and *TGF-β1.* On day 7, the gene expression levels of *CXCL-12* and *TGF-β1* were significantly elevated following S-PRG cement stimulation, compared to stimulation with MTA and controls (*p* < 0.01, Figure 8A,B). The gene expression levels of *CXCL-12* and *TGF-β1* were not regulated by MTA.

## 4. Discussion

Regeneration of the injured dentin–pulp complex, following trauma or caries progression, is one of the main purposes of endodontic procedures [36]. Hydraulic calcium-silicate cement was originally developed as a root-end filing material [37] and was found to result in better clinical outcomes for pulp capping when compared to Ca(OH)_2_ [8]. However, it has limitations, which include a high cost, a long setting time, and tooth discoloration [38,39]. In this study, we assessed the use of another cement, S-PRG, investigating its ability to induce mineralized tissue beneath the pulp capping material and its biological role in pulpal repair.

Therefore, in the present study, the mineralization of tissue induced by S-PRG cement was first evaluated using SEM and μCT analysis with regard to microstructure, quality, and quantity. Microstructural analysis by SEM revealed that S-PRG cement induced the formation of tertiary dentin with a tubular structure (Figure 1A,C,E). Some reports have previously demonstrated that MTA also induces the formation of tertiary dentin with a tubular structure [40,41], but we did not observe tubular structures in tertiary dentin induced by MTA. However, it is clear that both cements can induce some defects in the reparative dentin on occasion. It will be important to investigate whether these defects affect long-term sealing. Based on the results of µCT analysis, tertiary DV and tertiary DMD had similar characteristics to those of MTA at 4 weeks after pulp capping (Figure 2C,D). These findings show that S-PRG cement exhibits properties similar to MTA. 

Currently, there is a lack of reports regarding ion distribution from S-PRG cement. Thus, the µXRF analysis focused on Sr and Si ions, which were expected to induce tissue mineralization. Based on the results of elemental setting, S-PRG cement and MTA appeared to induce tissue mineralization with Ca and P (Figure 3A,B). Ions released from S-PRG cement were transferred into the pulpal tissue beneath S-PRG cement. Si and Sr ions contributed to the induction of mineralization [22,23]. Thus, these ions may be involved in pulpal repair. Further studies are needed to confirm whether these ions behave similarly in hDPSCs.

The effects of S-PRG cement on cell cytotoxicity and proliferation in hDPSCs were examined in vitro (Figure 4). These results demonstrated that S-PRG cement had similar characteristics to those of MTA, including low cytotoxicity and the ability to induce proliferation. Co-GIC differs from S-PRG cement with respect to powder content, high cytotoxicity, and the ability to inhibit proliferation. It is notable that small differences produced opposing effects in vitro. These results are also consistent with those of a previous report, indicating that Co-GIC is unsuitable for direct pulp capping [13]. Although Co-GIC was initially cytotoxic, it demonstrated the same biocompatibility as MTA and S-PRG after 2 weeks of culture. Co-GIC did not induce tertiary dentin formation (Figure S1). This means that the cytotoxicity (biocompatibility) of the direct pulp capping material is an important factor in tertiary dentin formation. Additionally, S-PRG cement has only been previously reported as a direct capping agent in a short-term observational in vivo study [25]. Long-term material stability and cytotoxicity assays using in vitro and in vivo evaluation are needed to assess its suitability for future clinical applications. After co-culture with an S-PRG disc, precipitates due to ion release were confirmed from S-PRG cement around the disc, regardless of the presence of osteoinductive media. Moreover, the precipitates were not present at cell-free sites in SEM analyses (Figure 5C). Representative images of μXRF could identify Ca and P around S-PRG discs. This result revealed that precipitates around S-PRG cement were derived from hDPSCs, rather than nonspecific precipitation, because these elements were not released from S-PRG cement. However, the precise nature of the interaction between hDPSCs and the material disk remains unclear. Further investigation of the morphological structure using time lapse imaging, and changes to hDPSCs at early stages using SEM observation, are necessary.

**Figure 9:**
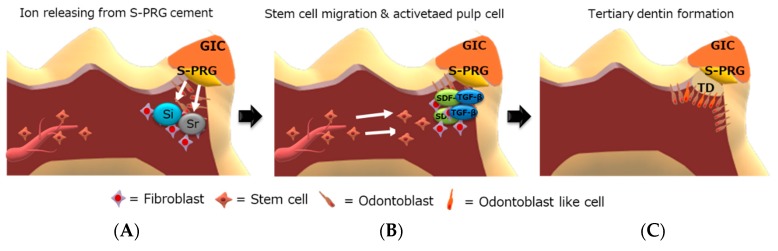
Proposed model for the mechanism by which surface pre-reacted glass-ionomer (S-PRG) acts on the pulpal wound healing process. After direct pulp capping, the high biocompatibility of S-PRG does not elicit an abnormal inflammatory response. Released ions (Sr and Si) from S-PRG diffuse into the pulp tissue (**A**). Pulp cells upregulate the expression of genes related to the osteo/dentinogenic differentiation process (*CXCL-12* and *TGF-β1*). *CXCL-12* (SDF-1) may also contribute to stem cell migration and promote wound healing (**B**). As a result of these processes, tertiary dentin is formed beneath S-PRG (**C**). S-PRG = S-PRG cements, GIC = Glass-ionomer cement, and TD = Tertiary dentin.

Next, RNA sequence analysis was performed to evaluate the effects of S-PRG cement on the global and osteo/dentinogenic gene expression in hDPSCs. These findings showed that *S*-PRG cement and MTA contributed to the formation of tertiary dentin through different mechanisms. We focused on *CXCL-12* and *TGF-β1*, which are related to osteo/dentinogenic mechanisms. Both *CXCL-12* and *TGF-β1* were regulated by S-PRG cement, but not by MTA (*p* < 0.01, Figure 8A,B). *CXCL-12* is widely known to promote regeneration and wound healing processes in several tissues [42,43], and promotes differentiation in dental pulp tissue [44,45]. *TGF-β1* is also involved in the healing/regeneration processes of dental pulp in response to injury [46]. Indeed, there is evidence in the literature suggesting that the combination of *TGF-β1* and an appropriate delivery system could enhance tertiary dentin formation with fewer initial inflammatory responses in animal models of direct capping [47]. Based on our findings, the regulation of gene expression by S-PRG cement may contribute to the wound healing process within the dentin–pulp complex (Figure 9). Other genes may also be regulated by S-PRG during this process. To further expand this research, many genes and signaling pathways should be analyzed in future experiments. It may be of particular interest to investigate the expression of other genes associated with osteo/dentinogenic differentiation, such as *DSPP*, *Col-1*, *DMP*, and *RUNX2*. Additionally, there appear to be no reports of whole gene expression in pulp cells with direct pulp capping material interaction. Raw reads were deposited into the DDBJ sequence read archive (DRA, accession number: DRA008317). This dataset could have an important role in novel direct pulp capping development in the future.

The results demonstrate that S-PRG cement exhibits similar behavior to that of MTA, but that the mechanisms of wound healing may differ between these materials. Importantly, these findings do not indicate the superiority or inferiority of S-PRG cement or MTA. The gene expression analyses suggest that S-PRG cement comparatively promotes the osteo/dentinogenic differentiation of hDPSCs, which is consistent with the results of the mineralized formation assay in vitro. However, there was no significant difference in the quality or quantity of tertiary dentin induced by both cements in vivo. This may relate to the standardized cavity in the rat direct pulp capping model. Thus, a larger invasive model, such as a pulpectomy model or large-scale direct capping model in dogs, may be appropriate, and further studies are needed. This study had some limitations. For example, due to ethical considerations and costs, we did not assess the extent of tertiary dentin formation in the absence of a capping agent. However, we did observe that tertiary dentin does not form in the presence of Co-GIC, which suggests that its formation requires the presence of an appropriate capping agent.

MTA has excellent mechanical strength and shows alkalinity after setting [48]; moreover, it can be used as a device to gradually release Ca ions [49]. Eluates containing Ca ions from MTA have been shown to promote cell proliferation and migration of mesenchymal stem cells and pulpal tissue via the c-Jun N-terminal kinase (JNK), extracellular signal-regulated kinase (ERK), Mitogen-activated Protein Kinase (MAPK), and fibroblast growth factors receptor (FGFR)-mediated signaling pathways [50,51]. It is unclear which signaling pathway is modulated by S-PRG cement during the process of pulpal healing. Further detailed experiments involving these materials may reveal how they induce wound healing in dental pulp. 

## 5. Conclusions

S-PRG cement had similar effects to those of hydraulic calcium-silicate cement with regard to the quality and quantity of tertiary dentin, and biocompatibility of hDPSCs. *CXCL-12* and *TGF-β1* were upregulated following ion release from S-PRG cement and may contribute to tertiary dentin formation during the healing process in pulpal tissue. These findings suggest that S-PRG cement is a bioactive material that may be useful in not only direct pulp capping but also as a tool to elucidate the mechanism of the wound healing process in pulp tissue. 

## Figures and Tables

**Table 1:** Material proportions.

	Materials	Main Composition
MTA	hydraulic calcium-silicate cement (ProRoot mineral trioxide aggregate (MTA), Dentsply Sirona)	Powder: white tricalcium silicate, dicalcium silicate, calcium sulfate, silica, and bismuth oxide trioxide aggregate. Lot number 108824.
S-PRG	Surface pre-reacted glass-ionomer filler cement (Shofu)	Powder: surface pre-reacted glass-ionomer filler (fluoroboroaluminosilicate glass). Liquid: copolymer of acrylic acid, tricarboxylic acid, water, and others.
Co-GIC	Conventional glass-ionomer cement (Base cement, Shofu)	Powder: fluoroaluminosilicate glass, others. Lot number 071797. Liquid: copolymer of acrylic acid and tricarboxylic acid, tartaric acid. Lot number 061730.

**Table 2:** Summary of primer sequences used.

Name	5′ Sequence (Forward)	3′ Sequence (Reverse)
*CXCL-12*	GAGCCAACGTCAAGCATCTCAA	TTTAGCTTCGGGTCAATGCCAC
*TGF b1*	CCCAGCATCTGCAAAGCTC	GTCAATGTACAGCTGCCGCA
*GAPDH*	AATCCCATCACCATCTTCCA	TGGACTCCACGACGTACTCA

## Supplementary Materials

The following are available online at https://www.mdpi.com/2077-0383/8/9/1440/s1, Figure S1: Representative histological images (H–E staining) after direct pulp capping using conventional glass-ionomaer cement (Co-GIC) in 4-week samples.
