# Supplementary material for: Surface Pre-Reacted Glass Filler Contributes to Tertiary Dentin Formation through a Mechanism Different Than That of Hydraulic Calcium-Silicate Cement
Source: J Clin Med. 2019 Sep 11;8(9):1440. doi: 10.3390/jcm8091440 (PMC6780685; doi:10.3390/jcm8091440)
Supplement: Supplementary file 1 [file jcm-08-01440-s001.pdf]

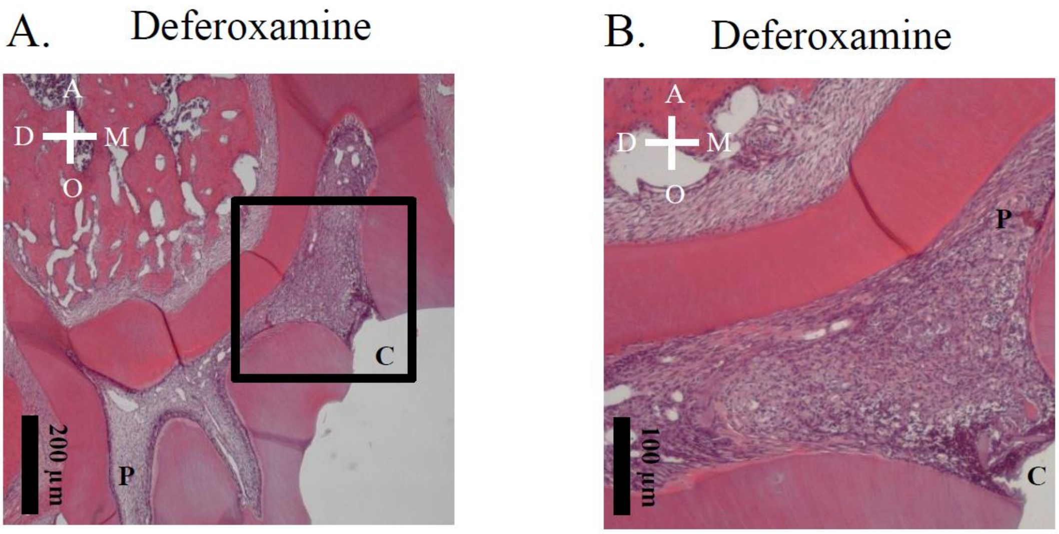

Figure S1. Representative histological images (H–E staining) after direct pulp capping using conventional glass-ionomaer cement (Co-GIC) in 4-week samples. Sagittal sections (**A**) and magnified views of insets (**B**) are shown. Mineralized tissue was not induced beneath cavity. This result is consistent with the fact that CO-GIC is not accepted as a direct capping material. C = cavity, P = pulp, D = distal, M = mesial, O = occlusal, A = apical.
